# Use of Surveillance Data on HIV Diagnoses with HIV-Related Symptoms to Estimate the Number of People Living with Undiagnosed HIV in Need of Antiretroviral Therapy

**DOI:** 10.1371/journal.pone.0121992

**Published:** 2015-03-13

**Authors:** Rebecca K. Lodwick, Fumiyo Nakagawa, Ard van Sighem, Caroline A. Sabin, Andrew N. Phillips

**Affiliations:** 1 Research Department of Primary Care and Population Health, University College London, London, United Kingdom; 2 Research Department of Infection and Population Health, University College London, London, United Kingdom; 3 Stichting HIV Monitoring, Amsterdam, Netherlands; FIOCRUZ, BRAZIL

## Abstract

**Background:**

It is important to have methods available to estimate the number of people who have undiagnosed HIV and are in need of antiretroviral therapy (ART).

**Methods:**

The method uses the concept that a predictable level of occurrence of AIDS or other HIV-related clinical symptoms which lead to presentation for care, and hence diagnosis of HIV, arises in undiagnosed people with a given CD4 count. The method requires surveillance data on numbers of new HIV diagnoses with HIV-related symptoms, and the CD4 count at diagnosis. The CD4 count-specific rate at which HIV-related symptoms develop are estimated from cohort data. 95% confidence intervals can be constructed using a simple simulation method.

**Results:**

For example, if there were 13 HIV diagnoses with HIV-related symptoms made in one year with CD4 count at diagnosis between 150–199 cells/mm^3^, then since the CD4 count-specific rate of HIV-related symptoms is estimated as 0.216 per person-year, the estimated number of person years lived in people with undiagnosed HIV with CD4 count 150–199 cells/mm3 is 13/0.216 = 60 (95% confidence interval: 29–100), which is considered an estimate of the number of people living with undiagnosed HIV in this CD4 count stratum.

**Conclusions:**

The method is straightforward to implement within a short period once a surveillance system of all new HIV diagnoses, collecting data on HIV-related symptoms at diagnosis, is in place and is most suitable for estimating the number of undiagnosed people with CD4 count <200 cells/mm3 due to the low rate of developing HIV-related symptoms at higher CD4 counts. A potential source of bias is under-diagnosis and under-reporting of diagnoses with HIV-related symptoms. Although this method has limitations as with all approaches, it is important for prompting increased efforts to identify undiagnosed people, particularly those with low CD4 count, and for informing levels of unmet need for ART.

## Introduction

Estimates of the number of people in a country or region who have undiagnosed HIV are important as they may prompt increased efforts to identify and treat such people, and may inform plans for the future delivery of antiretroviral therapy (ART). It is of particular importance to estimate the number of people with undiagnosed HIV who have a low CD4 count (below 350 cells/mm^3^, but especially below 200 cells/mm^3^) as treatment guidelines state that ART should be started without delay in such people, due to the risk of clinical disease [1].

Various methods have been used to estimate the number of people living with HIV in a particular region or country [2]. These include methods that make use of HIV prevalence survey data [3–5] and methods that apply “back-calculation” techniques to case reporting data on HIV/AIDS diagnoses [6–10]. While generally designed to estimate the overall number of people with HIV, these methods can also be used specifically to estimate the number of people with undiagnosed HIV. Although these methods have many advantages, they also have some limitations. Prevalence survey-based methods are not always straightforward to perform or interpret, especially when they are used to understand the CD4 count profile of a population. Back-calculation methods are often statistically complex and require reliable information on diagnoses and deaths in people with HIV in all preceding years.

In this paper, we describe an additional alternative method to estimate the number of people living with undiagnosed HIV with low CD4 count.

## Method

The method is based on the assumption that people with undiagnosed HIV who develop AIDS or other HIV-related symptoms of sufficient severity, or which are sufficiently specific to HIV, will present for care and be diagnosed with HIV as a result. This is essentially the same principle on which original back-calculation methods were based [11,12]. We will refer to these HIV-related symptoms which are likely to lead to presentation for care and HIV diagnosis simply as “HIV-related symptoms”. Such symptoms would typically refer to those listed among category B and C conditions (CDC-B and -C events) in the 1993 revised CDC classification system [13]. Only symptoms which are assumed to be caused by HIV are of interest, so symptoms related to a bacterial sexually transmitted infection, for example, should not count.

The data which are required for this method include data on the number of HIV diagnoses for which presence of HIV-related symptoms was a reason for the HIV test leading to diagnosis and the CD4 count at the time of diagnosis. It should be noted that these data requirements do not mean that all new diagnoses need to be specifically investigated for HIV-related symptoms (such as by additional physical examination), so collection of such information on all new HIV diagnoses should be feasible in any setting. Further, HIV diagnoses that are made due to symptoms of primary HIV infection should not be included.

**Fig 1:**
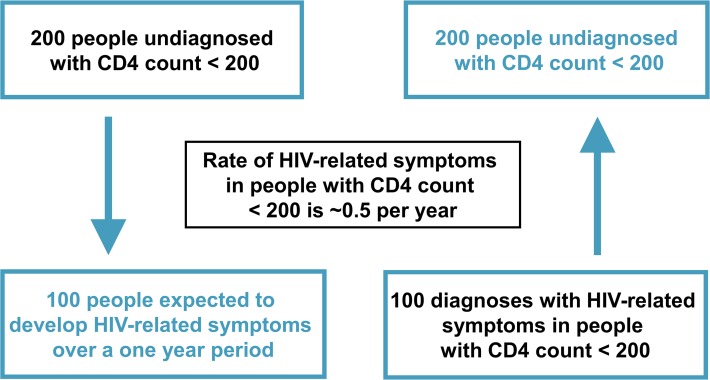
The basic concept underlying our method.

The main concepts of the method are demonstrated in Fig. 1 using the CD4 count stratum <200 cells/mm^3^ and are described as follows. The number of people who are diagnosed with HIV with HIV-related symptoms in a given CD4 count stratum over a specified period of time represents a proportion of the total undiagnosed population with CD4 count in that stratum. The size of the proportion is determined by the CD4 count-specific rate of such symptoms. Our method then reverses this logic: for each CD4 count stratum, the number of person-years lived with undiagnosed HIV is obtained by dividing the number of HIV diagnoses with HIV-related symptoms over a given period of time by the CD4 count-specific rate of HIV-related symptoms for that stratum. We thus consider this an estimate of the number of people living with undiagnosed HIV in the stratum. These estimates are then summed across the CD4 count strata to obtain an estimate of the total number of people with undiagnosed HIV within a broader CD4 count range.

The method requires knowledge of the CD4 count-specific rate of HIV-related symptoms of sufficient severity to lead to presentation and HIV diagnosis. While the CD4 count-specific rate of occurrence of AIDS is known, the CD4 count-specific rate of occurrence of such HIV-related symptoms is less well described. There is evidence in the literature to suggest that the rate of developing HIV-related symptoms is approximately two- to four-fold higher compared to the rate of AIDS[14–16]. In observational cohorts of people with HIV who are being followed for the presence of symptoms, more minor and less specific symptoms are likely to be identified: these are symptoms that would not necessarily lead to testing and consequent diagnosis in an undiagnosed person. In the absence of CD4 count-specific estimates for the rate of HIV-related symptoms, we therefore assume that the CD4 count-specific rates of HIV-related symptoms are two-fold the CD4 count-specific rates of AIDS (see Table 1). The AIDS rates were derived from data on the CASCADE collaboration of seroconverter cohorts [17,18]. They were calculated by counting the number of AIDS events to have occurred within a period of person-time, according to the most recent CD4 count measurement.

**Table 1:** Example calculations.

CD4 count *cells/mm* ^*3*^	*Estimated incidence rate of HIV- related symptoms per person-year*	*Estimated standard error of HIV- related symptoms*	*Example calculation for point estimate*		*Example calculation for confidence interval*	*Example calculation for high estimate*	
			*Number of observed HIV diagnoses with HIV-related symptoms in a year*	*Point estimate for the estimated number of person years lived with undiagnosed HIV in 2011 in stratum*	*95% confidence interval for the estimated number of person years lived with undiagnosed HIV in 2011 in stratum*	*Number of observed HIV diagnoses in a year*	*High estimate for the estimated number of person years lived with undiagnosed HIV in 2011 in stratum*
0–19	4.030	0.582	5	1	0–3	9	2
20–49	1.442	0.168	14	10	5–16	19	13
50–99	0.872	0.081	14	16	8–26	25	29
100–149	0.440	0.044	7	16	5–30	22	50
150–199	0.216	0.022	13	60	29–100	31	144
200–249	0.090	0.010	3	33^a^	0–80^a^	34	378^a^
250–299	0.076	0.007	3	39^a^	0–94^a^	41	539^a^
300–349	0.048	0.005	4	83^a^	18–176^a^	57	1188^a^
**Total (CD4 counts 0–200 cells/mm** ^**3**^)	-	-	**53**	**103**	**51–165**	**106**	**238**
**Total (CD4 counts 0–350 cells/mm** ^**3**^)	-	-	**63**	**258** ^a^	**159–415** ^a^	**238**	**2343** ^a^

Estimates for the number of person-years lived with undiagnosed HIV among MSM in the Netherlands in 2011, rounded to the nearest integer (not adjusted to take into account for missing CD4 counts or for reporting delay or under-reporting).

Note: Incidence rate and standard error of HIV-related symptoms obtained using data from the CASCADE cohort collaboration[16,17] (rate is two-fold the incidence of AIDS).

^a^These estimates should be interpreted with more caution due to the rates of HIV-related symptoms being much lower in the higher CD4 count ranges.

As rates of HIV-related symptoms at high CD4 counts (e.g. above 350 cells/mm^3^) are low, applying this method to HIV diagnoses occurring at higher CD4 counts would involve multiplying up each surveillance case by a very large number (the inverse of the rate of HIV-related symptoms) which may not produce stable estimates, albeit that this uncertainty is conveyed in the 95% confidence intervals (see below). This method is therefore most appropriate for use in estimating the numbers of undiagnosed people with HIV in the lower CD4 count range; i.e. those in most need of ART (CD4 count below 200 cells/mm^3^).

This method is based on ‘London method 1’ in an editorial review on methods for estimating the size of the undiagnosed population [2]. Here, we have revised the approach to be based on presence of HIV-related symptoms as a reason for the HIV test leading to diagnosis, rather than restricting to AIDS itself. The reason for this is concern that people will often present with pre-AIDS symptoms and so due to them being diagnosed and (unlike in the 1980s when the back-calculation method was originally developed) treated, will not develop an AIDS-defining condition. Thus AIDS (CDC-C events) alone is likely not sufficiently sensitive as the sentinel surveillance indicator.

### Adjustment of the estimate

There may be diagnoses with symptoms where the CD4 count is missing. The estimate obtained from this method should be divided by the proportion of all diagnoses with symptoms where the CD4 count is known. This adjustment depends on the assumption that the probability that the CD4 count is missing is independent of the actual CD4 count value, which may only be appropriate if the proportion of diagnoses with missing CD4 count is sufficiently small.

If there are known to be reporting delays or under-reporting of HIV cases with HIV-related symptoms, then the estimates can further be adjusted. For example, if there is believed to be 3% of cases missing due to reporting delay or under-reporting, then the estimate can be further multiplied by a factor of 1/0.97.

### 95% confidence intervals

To obtain 95% confidence intervals for the estimated number of people living with undiagnosed HIV, we suggest implementing a simple simulation method. There are two sources of uncertainty for the estimate: the stochastic uncertainty concerning the CD4 count-specific rate of symptoms and the stochastic uncertainty associated with the possibility that the observed number of HIV diagnoses with HIV-related symptoms may not correspond to the expected number based on the CD4 count-specific rate of symptoms. The rate of symptoms is assumed to vary according to a Normal distribution (standard errors presented in Table 1). The observed number of HIV diagnoses with HIV-related symptoms is assumed to vary according to a Poisson distribution. We re-run the estimation procedure 10,000 times, each time sampling the values of the rate of symptoms and the number of diagnoses with HIV related symptoms from these distributions simultaneously. The 2.5^th^ and 97.5^th^ percentile of the resulting estimated number with HIV are taken to be the limits of the 95% confidence interval.

### Upper limit of estimate

The 95% confidence intervals described above convey uncertainty due to stochastic effects. However, there are other sources of uncertainty. In particular, for some surveillance systems it is possible that there is significant under-reporting of symptoms present at HIV diagnosis, which would lead to under-estimation of the number living with undiagnosed HIV. We therefore also suggest calculation of an extreme upper limit for the number living with undiagnosed HIV with low CD4 count which is based on the assumption that all HIV diagnoses were made due to presence of symptoms.

## Results

The method is applied to surveillance data on all HIV diagnoses with HIV-related symptoms and CD4 count below 350 cells/mm^3^ in the country or region during the past year, grouped by CD4 count strata. The method is demonstrated using data from 2011 on HIV diagnoses in the Netherlands among men who have sex with men (MSM).

The calculation of the number with undiagnosed HIV and CD4 count below 350 cells/mm^3^ is presented in Table 1, showing a scenario in which there are a total of 63 HIV diagnoses with symptoms during one year. The estimated number with undiagnosed HIV is then calculated for each CD4 count stratum, for example 13/0.216 = 60 for CD4 count 150–199 cells/mm^3^. The overall estimate for the number of people living with undiagnosed HIV with CD4 count <350 cells/mm^3^ is then the sum of these, 258.

The overall estimate may then be adjusted to take into account the overall proportion of diagnoses with symptoms where the CD4 count is missing. In the Dutch data, 7% of all diagnoses with symptoms (including those with CD4 count at or above 350 cells/mm^3^) did not have a CD4 count at or near the time of diagnosis, so then the modified estimate would be 258/0.93 = 277. The estimate can be further adjusted to account for reporting delay or under-reporting, which is thought to be approximately 2% for the 2011 data. The estimate would then be further modified to 277/0.98 = 283.

The 95% confidence intervals for each CD4 count strata are also presented in Table 1. In this example, we used 10,000 simulation runs to obtain the limits. For the CD4 count stratum 150–199 cells/mm^3^, the 95% confidence interval is estimated to be 29–100 (95% confidence interval is 32–110 after adjustment for the proportion with missing CD4 count and under-reporting). The point estimate and 95% confidence interval for the total number of people living with undiagnosed HIV with CD4 count <200 and <350 cells/mm^3^ are 103 (51–165) and 258 (159–415) respectively. These are further adjusted to 113 (56–181) and 277 (171–455) respectively (results not shown in Table 1) to account for the proportion with missing CD4 count and under-reporting.

The extreme upper limit for the number with undiagnosed HIV and CD4 count below 350 cells/mm^3^, based on a situation in which all diagnoses of HIV are assumed to have resulted from symptoms is also included in Table 1. This gives a much higher value of 2343 (adjusted extreme upper limit is 2570).

## Discussion

The presented method uses surveillance data on HIV diagnoses with HIV-related symptoms to estimate the number of people living with undiagnosed HIV. The method allows estimation of the number of people with undiagnosed HIV in any given low CD4 count range, for example below 50 cells/mm^3^, where the need for ART is most urgent, or below 200 cells/mm^3^, which is the consensus cut-off used to define advanced HIV disease [19]. Although the method is most suitable for estimating the number with undiagnosed HIV with CD4 counts below 200 cells/mm^3^ in particular, it can also be used for estimating the number with undiagnosed HIV and CD4 count between 200 and 350 cells/mm^3^, but should be interpreted with more caution due to the rate of developing HIV-related symptoms being much lower.

Our method is more straightforward to implement than existing back-calculation methods and does not require the extensive data collection associated with methods based on prevalence survey methods. Consequently this method could be applied in resource-limited settings if the necessary data on all new diagnoses with HIV-related symptoms are collected for a period of time. It could also be used as an additional alternative method in high-income settings where such data already exist. The main advantage of our method is that only a limited period (e.g. one year) of accurately collected surveillance data on the number of people newly diagnosed with HIV who present with HIV-related symptoms, with their CD4 count, is required.

As the method works best when there are high levels of accurate ascertainment of HIV diagnoses with symptoms, a potential source of bias is the possible under-diagnosis and under-reporting of such diagnoses. Although AIDS is often notified and diagnosed along with HIV, audits have shown that there have been missed opportunities for HIV diagnoses where the patient presents with CDC-B and AIDS events[20]. On the other hand, the number of symptoms which occur during primary HIV infection may increase with increased awareness and campaigns over time, especially among certain high-risk groups such as MSM. Data on people with a diagnosis due to symptoms of primary HIV infection should not be included when using this method, although their inclusion should not impact greatly on any estimates if these diagnoses were in people with higher CD4 counts. Working to improve the completeness of data on the presence of symptoms at diagnosis is thus an important priority for surveillance in countries who wish to use this convenient method.

The method depends heavily on the availability of CD4 count at diagnosis, so although an adjustment can be made if a small proportion of surveillance cases have missing CD4 count, the method will be less reliable where a larger number of cases have no CD4 count data. This is because if the assumption that those with missing CD4 count are representative of those with a CD4 count is not met, the resultant bias is greater as the proportion with missing CD4 count increases. It is conceivable that missing CD4 counts are more likely to be from individuals with lower CD4 counts, because people with lower CD4 counts are those who are most ill and have the highest risk of death, perhaps before CD4 counts can be measured. This will therefore potentially lead to a lower estimate of the number of people with undiagnosed HIV. The extent to which this bias may exist needs to be assessed in a sensitivity analysis, where people who died rapidly such that a CD4 count could not be measured, are assumed to be in the lowest CD4 count category.

We recognise that the CD4 count-specific rate of HIV-related symptoms is derived somewhat arbitrarily by doubling the AIDS rate, although it was based on data from more than one study of carefully followed cohorts [14–16], including a study of our own [14]. In choosing this value of two-fold, we considered the possibility that the CD4 count-specific rate of AIDS in people with undiagnosed HIV is actually higher than that observed in seroconverter cohorts, as ART-naïve patients under care may have been treated with *Pneumocystis jirovecii* pneumonia prophylaxis.

We did not attempt to stratify CD4 count-specific rates of HIV related symptoms according to age or by other factors which are known to be associated with the progression of AIDS [21], although such a development could be incorporated to refine the estimates.

We chose to demonstrate this method using HIV data from the Netherlands for 2011 because it allowed us to compare our results with those estimated using an alternative method [22]. In the comparison study, it was estimated that there were 140 (95% confidence interval: 120–160) and 540 (470–600) MSM living with undiagnosed HIV with CD4 count <200 cells/mm^3^ and <350 cells/mm^3^ respectively in 2011. Using our method, we found that the point estimate and 95% confidence interval for the number of people living with undiagnosed HIV with CD4 count <200 and <350 cells/mm^3^ were 113 (56–181) and 277 (171–455) respectively (after adjustment for the proportion with missing CD4 count at diagnosis and under-reporting). Considering the difficulties with estimating the size of a hidden population and the fact that the estimates rely on different approaches and input data, the estimates are generally relatively close and the two together helps to consolidate a feeling of understanding the true picture compared with having either one alone available. It should be noted that since the 95% confidence interval of our estimate only conveys uncertainty due to stochastic effects it is not necessarily surprising that the alternative estimate (for the total number undiagnosed with CD4 count <350 cells/mm^3^) does not lie within this interval. In addition, the alternative estimate produced using a back-calculation method, does not use data on all HIV-related symptoms at diagnosis, only AIDS. The alternative estimate was well within the extreme upper limit for our estimate of 2570.

In conclusion, although like all approaches this method has limitations, it is straightforward to implement soon after a good surveillance system is put in place and does not rely on historical data. As such, this method is important for prompting enhanced surveillance activity and ultimately for establishing increased efforts to identify people with undiagnosed HIV, particularly those with low CD4 count, and for informing future delivery of ART. As a distinctly different approach to existing methods, it can also be used in conjunction with others to allow triangulation of estimates.
